# Oral Microbiota Alterations and Potential Salivary Biomarkers in Colorectal Cancer: A Next-Generation Sequencing Study

**DOI:** 10.3390/pathogens15010043

**Published:** 2025-12-30

**Authors:** Salih Maçin, Özben Özden, Rugıyya Samadzade, Esra Saylam, Nurullah Çiftçi, Uğur Arslan, Serdar Yormaz

**Affiliations:** 1 Department of Medical Microbiology, Faculty of Medicine, Selcuk University, Konya 42250, Turkey; salihmacin@selcuk.edu.tr (S.M.); rukiyesamadzade@gmail.com (R.S.); emeterelliyoz@gmail.com (E.S.); ciftcinurullah72@gmail.com (N.Ç.); drarslanugur@gmail.com (U.A.); 2 Institute of Health Sciences, Department of Medical Biotechnology, Acıbadem University, Istanbul 34752, Turkey; 3 Department of General Surgery, Faculty of Medicine, Selcuk University, Konya 42250, Turkey; serdaryormaz@selcuk.edu.tr

**Keywords:** bioinformatics, biomarker, biotechnology, colorectal cancer, oral microbiota, next-generation sequencing

## Abstract

Colorectal cancer (CRC) has a high mortality rate worldwide. Oral and intestinal microbiota members may have an effect on gastrointestinal tumors’ pathogenesis, particularly in CRC. Designed as a pilot study, this study’s aim was to investigate the relationship between CRC and oral microbiota and to identify potential biomarkers for CRC diagnosis. Saliva samples were collected from recently diagnosed CRC patients (*n* = 14) and healthy controls (*n* = 14) between March 2023 and December 2023. Microbiota (16S rRNA) analyses were conducted on these saliva samples using a next-generation sequencing method. Phylogenetic analyses, including alpha diversity, principal component analysis (PCA), principal coordinate analysis (PCoA), beta diversity, biomarker, and phenotype analyses, were conducted using the Qiime2 (Quantitative Insights Into Microbial Ecology) platform. Alpha diversity indices (Shannon: *p* = 0.78, Cho1: *p* = 0.28, Simpson: *p* = 0.81) showed no significant difference between CRC and control groups. Beta diversity analysis using Bray–Curtis PCoA indicated significant differences in the microbial community between the two groups (*p* = 0.003). Examination of OTU distributions revealed that the *Mycoplasmatota* phylum was undetectable in the oral microbiota of healthy controls but was significantly elevated in CRC patients (CRC: 0.13 ± 0.30, Control: 0.00 ± 0.00, *p* < 0.05). Additionally, *Metamycoplasma salivarium*, *Bacteroides intestinalis*, and *Pseudoprevotella muciniphila* were undetectable in healthy controls but significantly more prevalent in CRC patients (*p* < 0.05 for all three species). LEfSe analysis identified eight species with an LDA score > 2, *Granulicatella adiacens*, *Streptococcus thermophilus*, *Streptococcus gwangjuense*, *Capnocytophaga* sp. FDAARGOS_737, *Capnocytophaga gingivalis*, *Granulicatella elegans*, *Bacteroides intestinalis*, and *Pseudoprevotella muciniphila*, as potential biomarkers. The results of this study contribute critical evidence of the role of oral microbiota in the pathogenesis of colorectal cancer. Alterations in the microbiota suggest potential biomarkers in understanding the biological mechanisms underlying CRC and developing diagnostic and therapeutic strategies.

## 1. Introduction

Malignant neoplasms of the large intestine are commonly known as colorectal cancer (CRC). Several risk factors are associated with CRC, including smoking, excessive alcohol consumption, low vegetable intake, obesity, and physical inactivity. On the other hand, postmenopausal hormone use, nonsteroidal anti-inflammatory drug (NSAID) use, and high calcium intake are associated with a reduced risk of CRC. Despite these associations, the precise etiology of CRC remains unclear [1,2]. When detected at an early stage, colorectal cancers can be effectively treated with a high cure rate through appropriate surgical interventions. With proper patient management, including pathology review and adjuvant therapy following surgery, morbidity and mortality rates can remain minimal [3]. Approximately 20% of colon cancer cases are familial, and first-degree relatives of these patients have an elevated risk of developing CRC [4].

The human microbiota comprises a community of bacteria, fungi, viruses, archaea, protozoa, and other microorganisms, whose composition varies based on environmental conditions. Recent studies have highlighted the role of microbiota in carcinogenesis [5]. In addition to contributing to carcinogenesis, microbiota influences general cancer susceptibility, disease progression, coinfections, and responses to anticancer therapies [6].

The oral microbiota, which encompasses the microbial communities of the human oral cavity, is highly diverse, comprising over 700 distinct bacterial species. Due to its proximity to multiple anatomical sites, it is one of the most abundant microbiomes in the human body, second only to the colonic microbiota [7]. Recent studies have investigated the presence of various bacterial species in the oral microbiota and intestinal flora in gastrointestinal tumors, particularly CRC. These studies emphasize the significance of the oral microbiota in CRC [8].

Studies investigating the interaction between CRC and the oral microbiota have hypothesized a potential role for oral microbiota in CRC carcinogenesis. Several studies support this hypothesis by demonstrating the critical role of the oral microbiota in cancer development [9]. This study aimed to compare the oral microbial diversity of CRC patients with healthy controls, identify the oral microbiota profiles of CRC patients, investigate the association between CRC and oral microbiota, and detect potential microbial differences and biomarkers that can indicate or differentiate for CRC.

## 2. Materials and Methods

Fourteen patients diagnosed with colorectal cancer (CRC) between March 2023 and December 2023 at the General Surgery Outpatient Clinic, who had not yet received treatment, were included in the study. As a control group, 14 healthy individuals who tested negative in CRC screening were included.

The inclusion criteria for the study were as follows:

- Participants aged over 18 years.
- Non-pregnant individuals.
- No antibiotic use in the past three months.
- No recent surgical interventions.
- Absence of chronic diseases or active infections.

### 2.1. Microbiota Analysis

#### 2.1.1. Sample Collection and Storage

Saliva samples were collected in sterile Falcon tubes, labeled appropriately, and stored at −80 °C until sequencing.

#### 2.1.2. DNA Isolation from Saliva Samples

Genomic DNA was extracted from saliva samples using the Quick-DNA™ Fecal/Soil Microbe Miniprep Kit (Zymo Research, CA, USA) according to the manufacturer’s protocol.

Briefly, approximately 200 µL of each sample was mixed with Proteinase K and lysis buffer and incubated at 56 °C to ensure efficient degradation of mucins and cellular proteins.

After mechanical and enzymatic lysis, DNA was purified through a silica-membrane column, eluted in 100 µL of buffer, and stored at –20 °C until sequencing.

#### 2.1.3. Amplification of 16S Hypervariable Regions

2 µL of isolated DNA was taken and transferred into clean PCR tubes. Deionized water (ddH2O) was added to bring the final volume to 8 µL. 2 µL of 16S primer pair was added in tubes. The primer pair used targets a region of approximately 1500 bp that spans the V1-V9 regions of the 16S rRNA gene. The16S primer sequences; Forward Primer, 27F, *AGAGTTTGATCMTGGCTCAG*; Reverse Primer, 1492R, CGGTTACCTTGTTACGACTT.

Then, 10 µL of HF515 2x HiFi Taq Master Mix (MobiomX) enzyme was pipetted into each tube. The tube contents were gently mixed and briefly spun to collect any liquid from the tube walls at the bottom. The prepared samples were loaded into a MyGeneTM L Series Peltier Thermal Cycler (LongGene, China), set for thermal cycling conditions. 3 µL of the PCR-amplified products were taken and run on a 1% agarose gel at 100 V for 30 min. Amplicon sizes were confirmed in the range of 1–1.5 kb.

#### 2.1.4. Library Preparation and Sequencing

Following PCR amplification, sequencing was performed using Oxford Nanopore Technology (Oxford Nanopore Technologies, UK), a third-generation long-read sequencing platform.

The platform determines DNA sequences by detecting electrophysiological changes as DNA molecules pass through nanopores, allowing rapid library preparation and real-time long-read sequencing.

Amplicons were prepared using the ligation-based library preparation kit (SQK-NBD114.96) and sequenced on FLO-MIN114 (R10.4.1) flow cells with the Mk1C sequencer (Oxford Nanopore Technologies, UK).

Library preparation included DNA end repair, barcode ligation, and adapter ligation, followed by magnetic bead purification according to the manufacturer’s instructions.

The final DNA library concentration was measured using a Qubit fluorometer (Thermo Fisher Scientific, USA) before loading.

Sequencing was continued until a minimum of 10,000 reads per sample was obtained.The Oxford Nanopore long-read system was chosen for its ability to sequence the entire 16S gene and provide greater genome coverage and species-level taxonomic resolution compared with short-read platforms such as Illumina.

### 2.2. Bioinformatics Analysis

After sequencing, the results obtained in fast5 format were converted to fastq format using the Guppy software (version 6.5.7) (base-calling and de-multiplexing). Taxonomic assignment of 16S rRNA sequences was performed using the SILVA v138 reference database. Chimeric sequences were identified and filtered using VSEARCH. Since the 16S rRNA region is approximately 1500 bp, reads ranging from 1250 to 1750 bp were filtered using Trimmomatic, and the remaining reads were excluded from the analysis. The cleaned reads were analyzed using a custom workflow implemented in Python (3.12) programming language. During the filtering process, each sequence was matched using the BLAST algorithm. Sequences with more than 60% reference coverage and 80% pairwise similarity were selected, and their taxonomic data were extracted to create Operational Taxonomic Units (OTUs).

The generated OTU (.biom) file was used for phylogenetic analyses in the Qiime2 platform, including alpha diversity, PCA, PCoA, beta diversity, biomarker identification, and phenotype analyses. Prior to alpha and beta diversity calculations, the sequence data were rarefied to 10,000 reads per sample to standardize sequencing depth across all samples. Potential batch effects were evaluated using PCoA and PERMANOVA. Sequencing quality metrics, including mean read length and Q-score distributions, were calculated for all samples. Taxonomic classifications were organized using the Mothur platform, and dynamic Krona charts were prepared for visualization. All graphics and tables were generated using Python libraries. The complete bioinformatics workflow, from raw sequencing reads to final statistical and biomarker analyses, is illustrated in Supplementary Figure S1.

### 2.3. Statistical Analysis

All statistical analyses were performed using R version 4.2.1 software. Numerical variables were presented as mean ± standard deviation, while categorical variables were presented as frequency (*n*) and percentage (%). The mean age of CRC patients and healthy controls were compared using Student’s *t*-test, and the gender distribution was compared using the Yates continuity-corrected chi-square test. Additionally, the presence of significant differences in the detection of taxa at the phylum, class, family, genus, and species levels between CRC patients and healthy controls was compared using the Mann–Whitney U test. A significance level of 5% was applied.

## 3. Results

### 3.1. Demographic Findings

The study included 14 CRC patients (7 women, 7 men) and 14 healthy controls (7 women, 7 men). The mean age of the CRC patients was 58.36 ± 13.07, while the mean age of the control group was 58 ± 14.78.

### 3.2. The Results of Oral Microbiota Analysis

#### 3.2.1. Alpha Diversity

**Figure 1:**
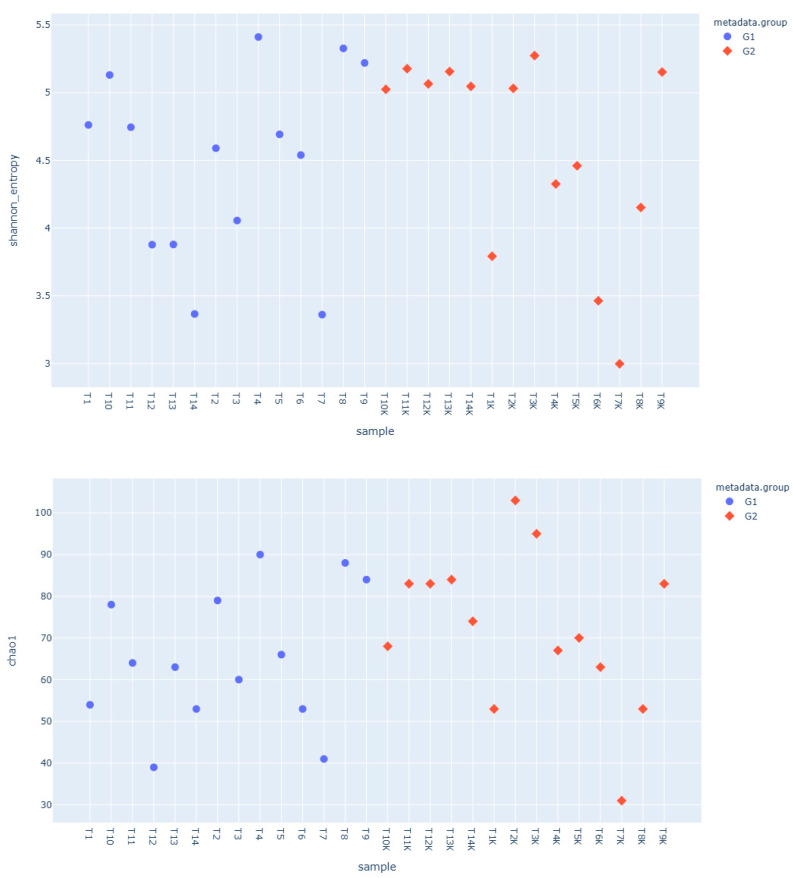

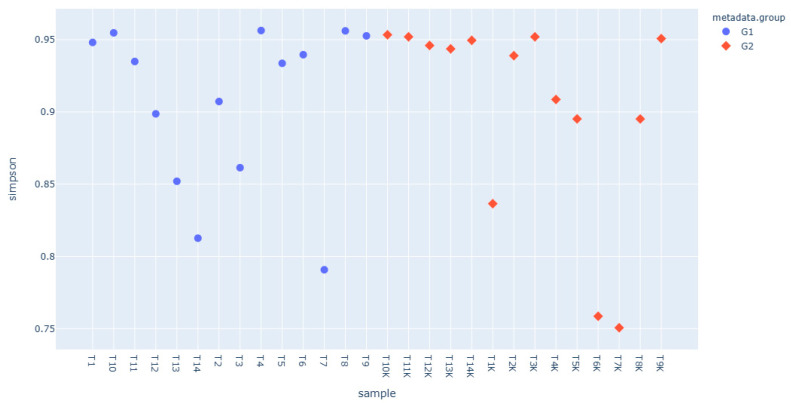
The Shannon, Choa1, and Simpson index distribution graph (G1: patients, G2: controls).

The alpha diversity of the saliva samples was calculated using the Shannon, Chao-1, and Simpson indices. No significant difference was found in the oral microbial alpha diversity between the groups for all three indices (*p* = 0.78, *p* = 0.28, *p* = 0.81, respectively) (Figure 1).

#### 3.2.2. Beta Diversity

**Figure 2:**
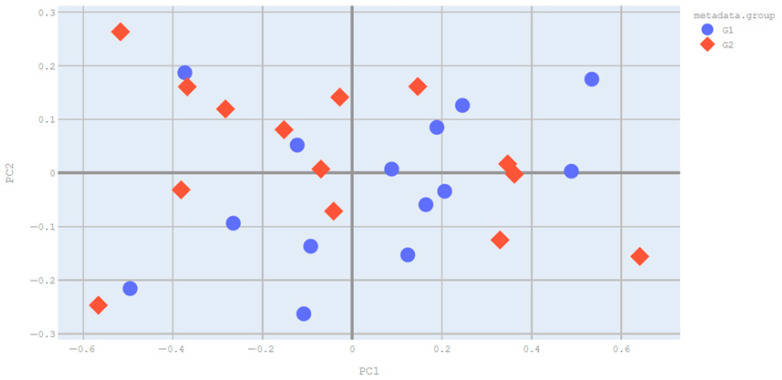
Bray–Curtis PCoA Analysis Plot (**G1:** patients, **G2:** control).

The beta diversity of the saliva samples was calculated using Bray–Curtis PCoA analysis. The Bray–Curtis PCoA beta diversity analysis showed that there was statistically significantly difference between the groups in terms of oral microbiota (*p* = 0.003) (Figure 2).

In addition, a Principal Component Analysis (PCA) was performed to further assess the distribution and homogeneity of the CRC and control groups. The PCA plot showed a partial separation between the two groups, consistent with the PCoA results (Supplementary Figure S2).

#### 3.2.3. Distribution of Operational Taxonomic Units (OTUs)

**Figure 3:**
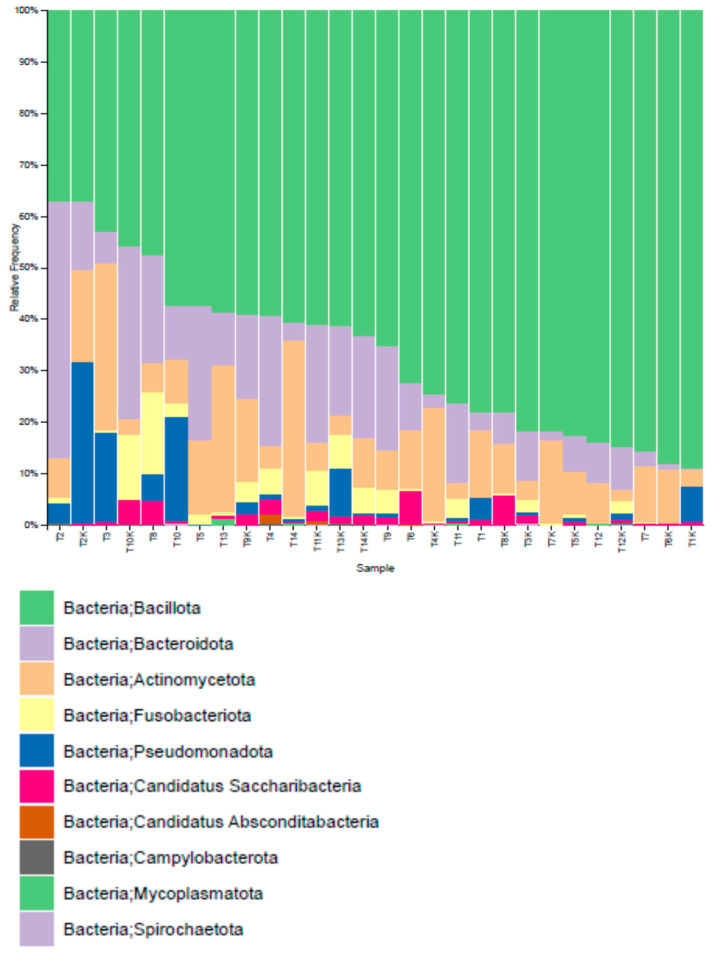
Microbiota Analysis at Phylum Level (GP1: patients, GP2: controls).

When the taxonomic distribution of OTUs in oral microbiota samples was examined at the phylum level, it was observed that both groups consisted of five main phyla: *Bacillota* (*Firmicutes*), *Bacteroidota*, *Actinomycetota*, *Pseudomonadota* (*Proteobacteria*), and *Fusobacteriota* (Figure 3).

The oral microbiota of healthy controls did not contain any *Mycoplasmatota* phylum, while a significantly higher presence of this phylum was observed in CRC patients (*p* = 0.038) (Table 1).

When the oral microbiota composition of the study groups was examined at the family level, *Carnobacteriaceae*, *Mycoplasmoidales*, and *Flavobacteriaceae* were found to be significantly higher in CRC patients compared to healthy controls (*p* = 0.021, *p* = 0.038, *p* = 0.007, respectively). The *Metamycoplasmataceae* family was not detected in healthy controls but was found to be significantly higher in CRC patients (*p* = 0.038) (Table 2).

When the oral microbiota composition of the study groups was examined at the genus level, *Granulicatella* and *Capnocytophaga* were found to be higher in CRC patients compared to healthy controls (*p* = 0.021, *p* = 0.038, respectively). The *Pseudoprevotella* genus was not detected in healthy controls, but it was found to be significantly higher in CRC patients (*p* = 0.038) (Table 3).

When the oral microbiota composition of the study groups was examined at the species level, *Granulicatella adiacens*, *Streptococcus thermophilus*, *Streptococcus gwangjuense*, *Capnocytophaga* spp. FDAARGOS_737, *Capnocytophaga gingivalis*, and *Granulicatella elegans* were found to be higher in CRC patients compared to healthy controls (*p* = 0.021, *p* = 0.05, *p =* 0.032, *p* ≤ 0.001, *p* = 0.03, *p* = 0.029, respectively). The species *Metamycoplasma salivarium*, *Bacteroides intestinalis*, and *Pseudoprevotella muciniphila* were not detected in healthy controls but were found to be significantly higher in CRC patients (*p* = 0.038, *p* = 0.038, *p* = 0.038, respectively) (Table 4).

### 3.3. LEFse Analysis

**Figure 4:**
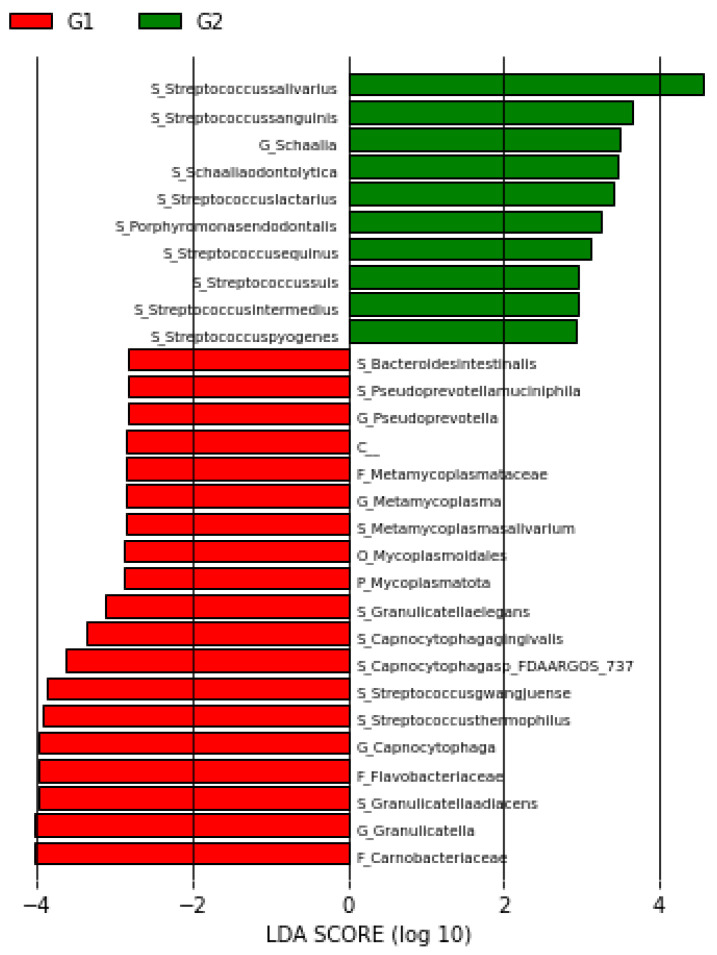
LEfSe plot of taxa at the genus and species levels (G1: patient group, G2: control group).

LEfSe (Linear Discriminant Analysis Effect Size) is an algorithm designed for biomarker discovery, which identifies significant differences between groups and determines genomic features associated with these differences. In LEfSe analysis, the Linear Discriminant Analysis (LDA) score is used as a measure of effect size. Taxa with an LDA score > 2 are considered potential biomarkers. In our study, the LEFse analysis identified eight different species with an LDA score > 2. These species were *Granulicatella adiacens*, *Streptococcus thermophilus*, *Streptococcus gwangjuense*, *Capnocytophaga* sp. FDAARGOS_737, *Capnocytophaga gingivalis*, *Granulicatella elegans*, *Bacteroides intestinalis*, and *Pseudoprevotella muciniphila* (Figure 4).

## 4. Conclusions

Colorectal cancer (CRC) accounts for 10% of all new cancer cases worldwide and is the third most common cancer type in men (after prostate and lung cancers) and in women (after breast and thyroid cancers). This high prevalence significantly increases the health burden on societies. Factors such as socioeconomic status, genetics, nutrition, and environmental influences affect CRC incidence and mortality rates [10]. In recent years, studies have suggested that the microbiota can contribute to both the initiation and progression of CRC. These studies indicate that changes in the intestinal microbiota during CRC development are associated with disease progression and diagnostic relevance [11].

Research investigating the association between oral microbiota diversity and colorectal cancer (CRC) has produced inconsistent results. While some studies reported no significant changes in α diversity [12,13], others—such as Rezasoltani et al.—demonstrated distinct clustering patterns in β diversity between healthy and CRC groups, indicating variations in community composition rather than overall microbial diversity [14]. These discrepancies may reflect population-specific factors, sampling sites, or methodological differences in sequencing depth and normalization approaches.

In this study, although α diversity indices (Shannon, Chao-1, Simpson) did not differ significantly between CRC and control groups, β diversity analysis revealed a clear compositional separation. This indicates that CRC is associated with qualitative, not quantitative, alterations in oral microbial communities. Such structural changes may enhance the abundance of specific taxa with pro-carcinogenic potential, such as Fusobacterium, *Porphyromonas*, and *Peptostreptococcus* species, which are known to promote chronic inflammation, epithelial barrier dysfunction, and modulation of immune responses [15,16,17].

Importantly, the observed enrichment of *Mycoplasma* spp. in CRC patients may highlight a previously underexplored mechanistic association. These bacteria can persist intracellularly, interfering with host cell apoptosis, DNA repair, and cell cycle regulation, thereby facilitating genomic instability and malignant transformation [18,19,20]. This study indicates that oral microbial dysbiosis is associated with CRC pathogenesis through modifications in microbial community structure and functional profiles, rather than through global diversity reduction.

Chronic inflammation and tumor progression are closely linked to p53 signaling, a central pathway in tumor suppression. Certain *Mycoplasma* species such as *M. fermentans*, *M. penetrans*, and *M. hyorhinis* have been reported to modulate p53 activity and reduce apoptosis in experimental models, suggesting possible interactions between persistent bacterial infection and host cellular regulation [21,22]. Prolonged *Mycoplasma* exposure in vitro has also been associated with phenotypic alterations such as chromosomal instability and abnormal cell growth [23].

In this study, members of the *Mycoplasmatota* phylum were detected exclusively in CRC patients and were absent in healthy controls (*p* = 0.038). While this finding may indicate a potential association, it should be interpreted with caution. The current data do not establish a direct causal relationship between *Mycoplasma* and CRC. Instead, these bacteria may reflect secondary changes in the tumor microenvironment or opportunistic colonization associated with host immune dysregulation. Further studies are required to determine whether *Mycoplasma* actively contributes to carcinogenesis or merely represents a microbial biomarker associated with CRC.

Large cohort studies have also explored the relationship between oral microbiota and CRC. For instance, 16S rRNA gene sequencing of saliva samples identified oral taxa such as *Bifidobacteriaceae*, *Prevotellaceae*, and *Carnobacteriaceae* as being positively associated with CRC risk, suggesting that intestinal colonization may result from the translocation of oral microbes [24]. Similarly, a study in Iran identified *Bifidobacteriaceae* as a potential biomarker in the saliva of CRC patients [14]. Flemer et al. reported higher abundance of *Lachnospiraceae* in the salivary microbiota of CRC patients compared to controls [25]. Idiopathic pulmonary fibrosis group using antifibrotic drugs and that not using antifibrotic drugs were compared, only *Lachnospiraceae UCG 004* abundance was found to be lower in the patient group receiving antifibrotic drugs [26]. In this study, *Carnobacteriaceae*, *Mycoplasmoidales*, and *Flavobacteriaceae* were significantly elevated in CRC patients (*p* = 0.021, *p* = 0.038, *p* = 0.007, respectively).

Several oral taxa (*Streptococcus* spp. and *Prevotella* spp.) were detected in greater abundance in CRC patients compared with controls. This suggests that some microbiota species play a protective role against CRC, possibly by conferring colonization resistance to CRC-associated oral taxa [27]. In a similar study investigating the association between CRC and oral microbiota, *Fusobacterium* spp., *Dialister* spp., *Catonella* spp., *Tennerella* spp., *Eubacterium*-brachy-group and *Fretibacterium* spp. group bacteria have been identified as possible markers with potential to distinguish healthy individuals from CRC patients [28].

In this study, when the oral microbiota composition was examined at the genus level, *Granulicatella* and *Capnocytophaga* genera were detected more in CRC patients compared to healthy controls (*p* = 0.021, *p* = 0.038, respectively). It is also significant that the *Pseudoprevotella* genus was not detected in healthy controls but was detected at a significantly higher level in CRC patients (*p* = 0.038).

According to data from various studies, species such as *Granulicatella adiacens* and *Capnocytophaga gingivalis* are considered as pathogenic microorganisms associated with cancer [29,30,31]. In a study investigating the oral microbiota of lung cancer patients, the proportion of *Capnocytophaga* was found to be significantly higher in the saliva of patients compared to healthy controls [32,33]. Another study reported that *Capnocytophaga gingivalis* could be a potential biomarker in the saliva of oral cancer patients. These bacteria may contribute to carcinogenesis through several potential mechanisms, including the induction of chronic inflammation, production of genotoxic metabolites, and disruption of epithelial barrier integrity. Such interactions can promote a pro-tumor microenvironment, suggesting that *Granulicatella* and *Capnocytophaga* might play functional roles in colorectal cancer development [34].

In this study, when the oral microbiota composition was examined at the species level, *Granulicatella adiacens*, *Streptococcus thermophilus*, *Streptococcus gwangjuense*, *Capnocytophaga* sp. FDAARGOS_737, *Capnocytophaga gingivalis* and *Granulicatella elegans* species were detected higher in CRC patients compared to healthy controls (*p* = 0.021, *p* = 0.05, *p* = 0.032, *p ≤* 0.001, *p* = 0.03, *p* = 0.029, respectively). While *Metamycoplasma salivarium*, *Bacteroides intestinalis* and *Pseudoprevotella muciniphila* species were not detected in healthy controls, their significantly higher detection in CRC patients demonstrates the potential of these species as biomarkers (*p* = 0.038, *p* = 0.038, *p* = 0.038, respectively).

This study contributes to a better understanding of the role of the oral microbiota in CRC development and identifies potential non-invasive biomarkers for early detection. While the results are consistent with existing literature, they also highlight unique microbial associations with CRC. In this study, the possible non-invasive biomarker potential of the *Mycoplasmatota* phylum at the phylum level, the *Metamycoplasmataceae* family at the family level and the *Pseudoprevotella* genus at the genus level, which were not found in the control group but were detected at high levels in the CRC group, was determined. In addition, according to LEFse analysis, *Granulicatella adiacens*, *Streptococcus thermophilus*, *Streptococcus gwangjuense*, *Capnocytophaga* sp. FDAARGOS_737, *Capnocytophaga gingivalis*, *Granulicatella elegans*, *Bacteroides intestinalis* and *Pseudoprevotella muciniphila* species were found to be significantly higher in the CRC group, indicating that they may have a high potential for use as non-invasive biomarkers in the early stages of the disease.

Significant beta-diversity differences in the oral microbiota of CRC patients are consistent with recent analyses of stool microbiota from the same individuals. Saylam et al. (2025) [35] reported that fecal samples from these participants exhibited marked compositional changes and reduced alpha diversity compared to healthy controls (Shannon *p* = 0.045; Bray–Curtis PCoA *p* = 0.004). Although the saliva samples did not show a significant decrease in alpha diversity (Shannon, *p* = 0.78), both oral and fecal samples revealed distinct taxa enriched in CRC, including *Bacteroides intestinalis* and other potential biomarker species [35]. These results suggest that microbial dysbiosis associated with CRC may occur at multiple anatomical sites, reflecting systemic microbial alterations. Nonetheless, species-specific differences and the relatively small sample size highlight the need for further studies to explore functional links between oral and gut microbiota in CRC.

Studies investigating salivary and fecal microbiota have shown marked differences between individuals with colorectal polyps and healthy controls. Microbial diversity was found to increase in saliva but decrease in feces among patients with polyps, while oral taxa such as *Porphyromonas gingivalis* and *Fusobacterium nucleatum* were frequently identified as major contributors. Furthermore, the combination of salivary and fecal microbiota biomarkers improved diagnostic performance in distinguishing patients with colorectal polyps from healthy individuals. These results suggest that integrating oral and gut microbiota could enhance the accuracy of non-invasive approaches for early colorectal lesion detection [36].

This study has several limitations. First, the sample size was relatively small, and a validation cohort or cross-validation strategy was not applied, which may limit the statistical power and reproducibility of the identified potential biomarkers. Several confounding factors, including diet, smoking, alcohol consumption, and oral hygiene, were not fully controlled, and the study was conducted at a single center, potentially reducing generalizability. Second, the study relied solely on Nanopore-sequenced saliva samples collected in our laboratory, without comparison to publicly available datasets, and differences in extraction kits, sequencing depth, and metadata limited direct cross-study validation. These factors highlight the need for future studies integrating publicly available Nanopore oral microbiome datasets to validate and generalize our findings across different cohorts and technical platforms. Finally, only saliva samples were analyzed, which may not fully reflect microbial composition throughout the gastrointestinal tract, and mechanistic studies are needed to clarify causal links between specific oral taxa and colorectal cancer.

In summary, the results obtained from this study are expected to play an important role in identifying potential non-invasive biomarkers for colorectal cancer and in the development of novel therapeutic strategies. Moreover, such biomarkers could contribute to early diagnosis and generate positive impacts on health economics by enabling timely intervention. Finally, conducting future studies with larger sample sizes will be crucial to strengthen the accuracy and validity of these findings, to better understand the biological mechanisms of cancer, and to develop patentable diagnostic and therapeutic biomarkers.

## Figures and Tables

**Table 1:** Oral Microbiota Composition at Phylum Level.

Phylum (Relative Abundance %)	Study Groups		*p* Value
	Control (*n* = 14)	CRC (*n* = 14)	
*Bacillota (Firmicutes)*	70.84 ± 16.19	63.25 ± 14.85	*0.125*
*Bacteroidota*	11.38 ± 9.72	15.12 ± 12.78	*0.352*
*Pseudomonadota (Proteobacteria)*	4.08 ± 8.42	4.04 ± 6.63	*0.982*
*Fusobacteriota*	2.80 ± 3.65	2.48 ± 4.15	*0.982*
*Actinomycetota*	9.33 ± 6.40	13.53 ± 10.35	*0.306*
*Candidatus saccharibacteria*	1.38 ± 1.71	1.20 ± 1.91	*0.462*
*Candidatus absconditabacteria*	0.08 ± 0.20	0.16 ± 0.54	*0.705*
*Campylobacterota*	0.04 ± 0.08	0.05 ± 0.09	*0.931*
*Mycoplasmatota*	0.00 ± 0.00	0.13 ± 0.30	*<0.05*
*Spirochaetota*	0.07 ± 0.12	0.04 ± 0.11	*0.388*

Note: *p*-values were calculated using the Mann–Whitney U test. For consistency, values below 0.05 are reported either as exact or threshold values depending on rounding precision.

**Table 2:** Oral Microbiota Composition at Family-Level.

Family (Relative Abundance %)	Study Groups		*p* Value
	Control (*n* = 14)	CRC (*n* = 14)	
*Carnobacteriaceae*	2.47 ± 1.85	5.10 ± 4.01	*0.021*
*Mycoplasmoidales*	0.00 ± 0.0	0.13 ± 0.3	*<0.05*
*Flavobacteriaceae*	0.43 ± 0.5	2.47 ± 2.97	*0.007*
*Metamycoplasmataceae*	0.00 ± 0.0	0.13 ± 0.3	*<0.05*

Note: Mann–Whitney U test, *p* < 0.05 was considered significant.

**Table 3:** Oral Microbiota Composition at Genus Level.

Genus (Relative Abundance %)	Study Groups		*p* Value
	Control (*n* = 14)	CRC (*n* = 14)	
*Granulicatella*	2.47 ± 1.85	5.09 ± 3.99	*0.021*
*Streptococcus*	57.49 ± 18.11	49.55 ± 15.02	*0.194*
*Fusobacterium*	0.44 ± 0.61	0.35 ± 0.54	*0.539*
*Pseudoprevotella*	0.00 ± 0.00	0.10 ± 0.23	*<0.05*
*Capnocytophaga*	0.43 ± 0.50	2.47 ± 2.97	*0.007*
*Porphyromonas*	2.78 ± 4.77	1.65 ± 2.93	*0.836*
*Veillonella*	2.61 ± 2.73	2.76 ± 2.78	*0.571*
*Megasphaera*	0.03 ± 0.09	0.19 ± 0.54	*0.353*
*Haemophilus*	0.38 ± 0.53	2.10 ± 4.74	*0.452*

Note: Mann–Whitney U test, *p* < 0.05 was considered significant.

**Table 4:** Oral Microbiota Composition at Species-Level.

Species (Relative Abundance %)	Study Groups		*p* Value
	Control (*n* = 14)	CRC (*n* = 14)	
*Granulicatella adiacens*	2.16 ± 1.8	4.44 ± 3.68	*0.021*
*Streptococcus thermophilus*	1.85 ± 1.26	2.84 ± 6.55	*0.050*
*Streptococcus gwangjuense*	0.46 ± 0.43	2.26 ± 2.26	*<0.05*
*Capnocytophaga* sp. *FDAARGOS_737*	0.10 ± 0.24	0.96 ± 1.43	*<0.001*
*Capnocytophaga gingivalis*	0.16 ± 0.20	0.57 ± 0.61	*0.030*
*Granulicatella elegans*	0.31 ± 0.36	0.65 ± 0.59	*0.029*
*Metamycoplasma salivarium*	0.00 ± 0.00	0.13 ± 0.3	*<0.05*
*Bacteroides intestinalis*	0.00 ± 0.00	0.09 ± 0.23	*<0.05*
*Pseudoprevotella muciniphila*	0.00 ± 0.00	0.1 ± 0.23	*<0.05*
*Prevotella denticola*	0.04 ± 0.11	0.11 ± 0.25	*0.405*
*Fusobacterium nucleatum*	0.02 ± 0.08	0.02 ± 0.05	*0.638*
*Porphyromonas gingivalis*	0.12 ± 0.23	0.03 ± 0.08	*0.353*
*Haemophilus parainfluenzae*	0.34 ± 0.43	2.08 ± 4.73	*0.397*

Note: Mann–Whitney U test, *p* < 0.05 was considered significant.

## Data Availability

The original contributions presented in this study are included in the article/Supplementary Material. Further inquiries can be directed to the corresponding author.

## Supplementary Materials

The following supporting information can be downloaded at: https://www.mdpi.com/article/10.3390/pathogens15010043/s1.
