# Supplementary material for: Oral Microbiota Alterations and Potential Salivary Biomarkers in Colorectal Cancer: A Next-Generation Sequencing Study
Source: Pathogens. 2025 Dec 30;15(1):43. doi: 10.3390/pathogens15010043 (PMC12845062; doi:10.3390/pathogens15010043)
Supplement: Supplementary file 1 [file pathogens-15-00043-s001.zip › pathogens-3941937-supplementary.pdf]

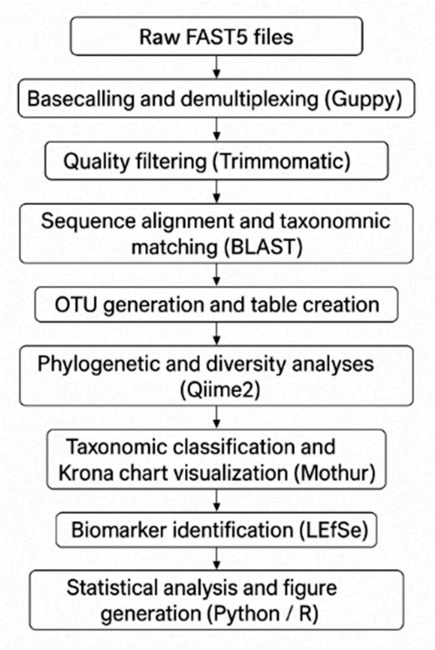

Supplementary Figure S1. Ioinformatics workflow used for microbiota data processing and analysis, illustrating the sequential steps from raw sequencing reads to final statistical and biomarker analyses.

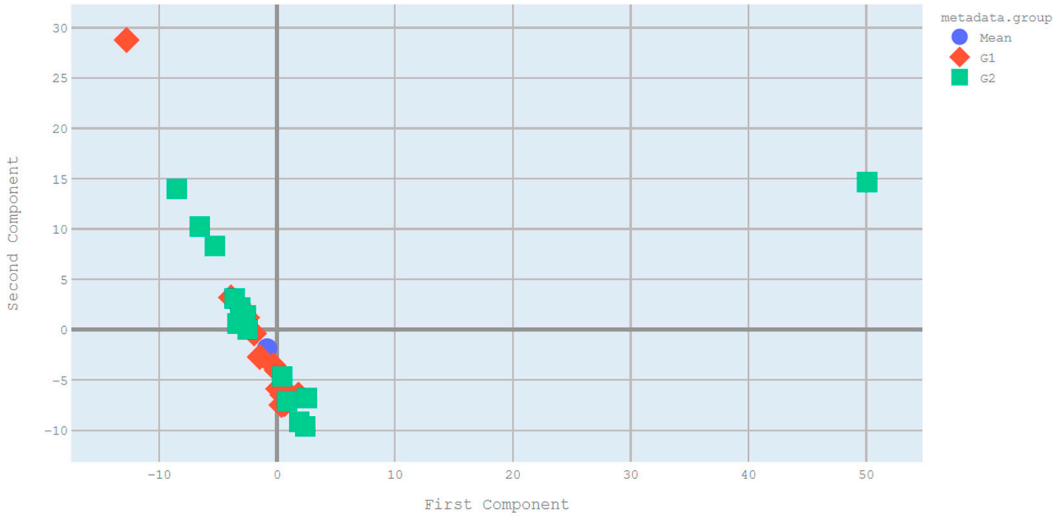

Supplementary Figure S2. Principal Component Analysis (PCA) plot of the samples.
